# Host Cell Responses to Persistent Mycoplasmas - Different Stages in Infection of HeLa Cells with *Mycoplasma hominis*

**DOI:** 10.1371/journal.pone.0054219

**Published:** 2013-01-11

**Authors:** Miriam Hopfe, René Deenen, Daniel Degrandi, Karl Köhrer, Birgit Henrich

**Affiliations:** 1 Institute of Medical Microbiology and Hospital Hygiene, Heinrich-Heine-University Düsseldorf, Düsseldorf, Germany; 2 Biological-Medical Research Centre, Heinrich-Heine-University Düsseldorf, Düsseldorf, Germany; University of Louisville, United States of America

## Abstract

*Mycoplasma hominis* is a facultative human pathogen primarily associated with bacterial vaginosis and pelvic inflammatory disease, but it is also able to spread to other sites, leading to arthritis or, in neonates, meningitis. With a minimal set of 537 annotated genes, *M. hominis* is the second smallest self-replicating mycoplasma and thus an ideal model organism for studying the effects of an infectious agent on its host more closely. *M. hominis* adherence, colonisation and invasion of HeLa cells were characterised in a time-course study using scanning electron microscopy, confocal microscopy and microarray-based analysis of the HeLa cell transcriptome. At 4 h post infection, cytoadherence of *M. hominis* to the HeLa cell surface was accompanied by differential regulation of 723 host genes (>2 fold change in expression). Genes associated with immune responses and signal transduction pathways were mainly affected and components involved in cell-cycle regulation, growth and death were highly upregulated. At 48 h post infection, when mycoplasma invasion started, 1588 host genes were differentially expressed and expression of genes for lysosome-specific proteins associated with bacterial lysis was detected. In a chronically infected HeLa cell line (2 weeks), the proportion of intracellular mycoplasmas reached a maximum of 10% and *M. hominis*-filled protrusions of the host cell membrane were seen by confocal microscopy, suggesting exocytotic dissemination. Of the 1972 regulated host genes, components of the ECM-receptor interaction pathway and phagosome-related integrins were markedly increased. The immune response was quite different to that at the beginning of infection, with a prominent induction of IL1B gene expression, affecting pathways of MAPK signalling, and genes connected with cytokine-cytokine interactions and apoptosis. These data show for the first time the complex, time-dependent reaction of the host directed at mycoplasmal clearance and the counter measures of this pestering pathogen.

## Introduction

*Mycoplasma hominis* is the second smallest, self-replicating mycoplasma species that colonizes humans. This facultative-pathogenic cell wall-less bacterium is found as a commensal in the urogenital tract of sexually active people, but is also associated with bacterial vaginosis, pelvic inflammatory disease, arthritis and even neonatal meningitis [1]. The patho-physiological mechanisms that enable this commensal to become pathogenic are mostly unresolved. In bacterial vaginosis shifts to a higher pH in vaginal flora are often accompanied by higher *M. hominis* titers. However, whether higher colonisation rates are the consequence or the reason for such changes in the milieu is still unknown.

For the last twenty years we have been interested in the characterisation of pathogenic factors of *M. hominis*. As attachment to host epithelial cells is thought to be the crucial step in infection, we began our studies with the identification of cytoadhesive membrane proteins, such as the P80 secretin and the lipoproteins P50/Vaa, P60 and OppA [2], [3], [4], and identified the multifunctional OppA protein as a pathogenic factor of *M. hominis*. Besides its function as the substrate-binding domain of an oligopeptide importer [5], OppA additionally induces ATP release and induces damage in host cells while its unique ecto-ATPase activity is a prerequisite for OppA-mediated cytoadhesion [6], [7]. The capacity of *M. hominis* to invade cells was firstly described in 1991 by Taylor-Robinson and coworkers, who used HeLa cells as host in an *in vitro* infection model [8]. Fifteen years later invasion into spermatozoa, leading to abnormal sperm morphology [9], was demonstrated [10]. With the detection of intracellular localisation and replication in another venereal pathogen, *Trichomonas vaginalis*, a symbiotic association between *T. vaginalis* (as Trojan horse) and *M. hominis* was elucidated [11]. This association was suggested to be a benefit for both, influencing the metronidazole susceptibility of the protozoan [12] and defending the invading mycoplasma from immune responses.

Detailed descriptions of the patho-physiological effects of a *M. hominis* infection on the host at different stages of infection (adhesion – invasion – survival) are still missing. Sequencing of the whole genome of the *M. hominis* type strain PG21 in 2009 led to the annotation of only 537 protein-encoding genes, of which 220 were predicted to be *M. hominis*-specific [13]. This reduced protein set suggests that *M. hominis* is an excellent model organism for studying host-pathogen interactions in detail. To study the cellular effects of a urogenital tract infection by *M. hominis* more closely, we established an *in vitro* infection model using the human cervix carcinoma cell line HeLa as host cell and the *M. hominis* isolate FBG as pathogen.

## Results

### Microscopic View of *M. hominis* Attachment to and Invasion in HeLa Cells

**Figure 1:**
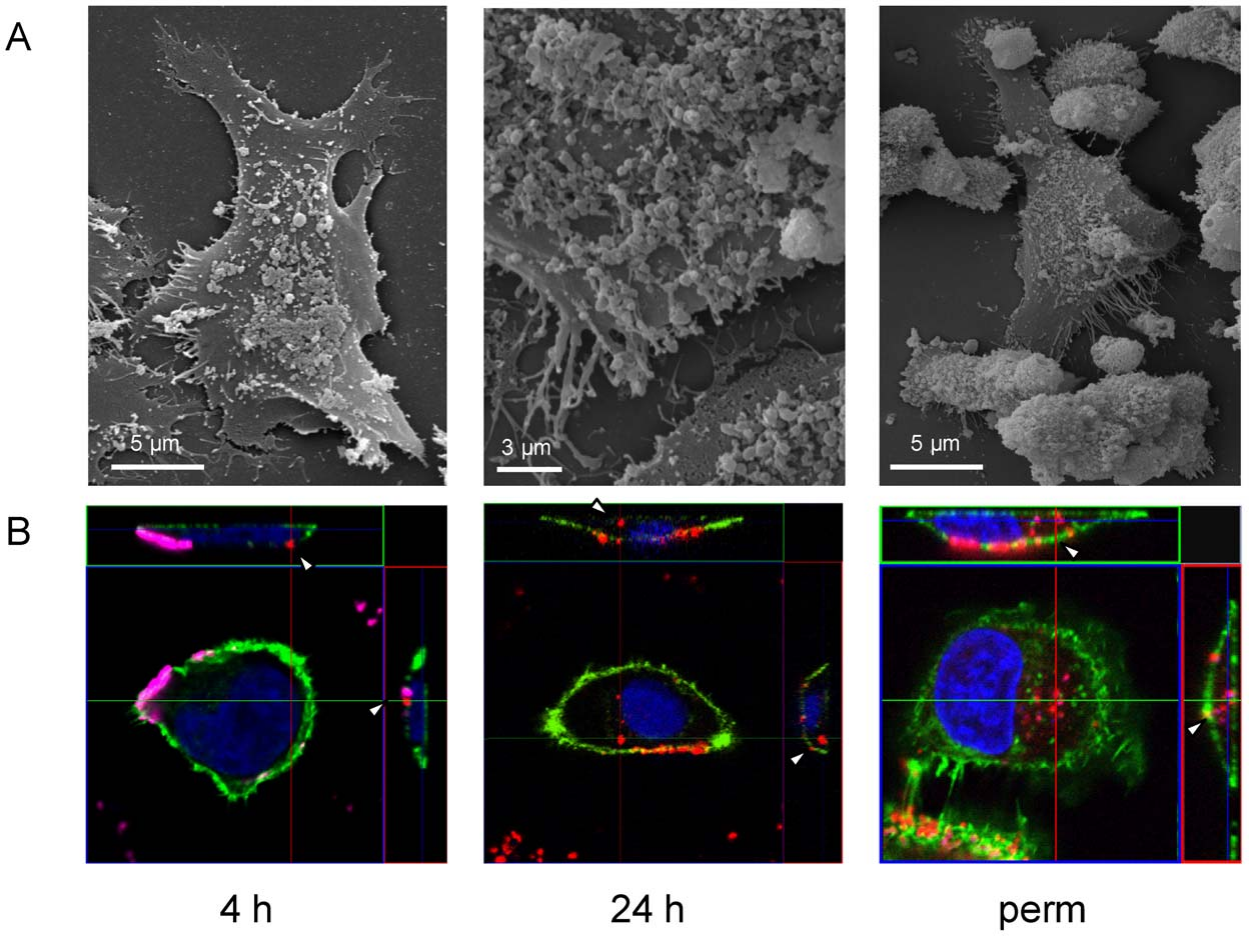
Microscopy of *M. hominis*-infected Hela cells. HeLa cells were analysed by scanning electron microscopy (A) and confocal laser microscopy (B) at 4 h, 24 h and 2 weeks (perm) post infection. Actin filaments were stained green (Alexa 488 phalloidin), nuclei blue (DAPI), surface-attached mycoplasmas magenta (B 4 h) or red (B 48 h and B perm) (mAb BG11 without fixation) and internalized mycoplasmas red (mAb BG11 after fixation and permeabilisation).

Initially, *M. hominis* adherence to and colonisation of HeLa cells were characterised over time, from 4 h to 2 weeks post infection, using scanning electron microscopy and confocal laser microscopy. As shown in Figure 1A, *M*. *hominis* cells attached to the glass-adherent HeLa cells preferentially on the convex side of the cell body (4 h) and then dispersed over the surface of the host cell. Colonisation led to a pronounced shortening of filopodia and contraction of the cell, which resulted in disruption of the cell monolayer (24 h). In a chronically infected cell line (i.e. 2 weeks post infection, perm) adherence of the infected HeLa cells to glass was less strong and the proportion of rounded host cells increased (Fig. 1A perm). In addition, empty HeLa shells with a hole in the membrane appeared. Cultivation of a *M. hominis*-infected HeLa cell line for more than 3 months did not lead to clearance of the pathogen.

Immunofluorescence studies using confocal microscopy confirmed these observations. As depicted in Figure 1B, actin microfilaments of the host, stained with Alexa Fluor 488 phalloidin (green), became condensed over the course of infection. The primarily surface-localised *M. hominis* cells (4 h post infection) were increasingly found intracellularly after 24 hours and were found predominantly in the cytoplasm of the chronically infected HeLa cells (perm). As shown in Figure 1B, *M*. *hominis* cells mainly adhered to the HeLa cell surface (depicted in magenta) and in only a few cases could mycoplasmal invasion be observed at this early stage of infection (4 h) (shown in red and marked by an arrowhead), at which three-fourth of all HeLa cells were colonised by *M. hominis*. To differentiate between intra- and extracellular mycoplasmal cells, staining was performed with a mycoplasma-specific antibody (BG11) without permeabilization and fixation of the cells, thus staining the surface-localised mycoplasmas. After fixation with paraformaldehyde and permeabilization of the host cells with Triton-X-100, staining of extra- and intracellular mycoplasma cells was performed. Mycoplasmas localised on the surface were coloured magenta and mycoplasmas residing inside the host cell are shown in red. Loosely bound HeLa cell shells were removed by this procedure. As seen in an overlay (yellow) of actin- (green) and mycoplasma- (red) staining, mycoplasma entry into the host cell seems to occur at and affect the cytoskeleton (Fig. 1B, 24 h). The ratio of intracellular and extracellular mycoplasmas, using the gentamicin assay as described by Winner et al. (2000) [14], indicated that 0.5% of the mycoplasmas appeared to be intracellular 24–48 h after infection. Thus, based on the 30-fold excess of *M. hominis* to HeLa cells (see Figure S1) and a colonisation rate of the HeLa cells of 95%, one-sixth of the HeLa cells should carry intracellular mycoplasmas at 48 h post infection.

As seen in confocal microscopy and calculated by qPCR, nearly all HeLa cells of the chronically infected HeLa cell line were colonised by a 50-fold excess of mycoplasma cells, 10% of which reside intracellularly as estimated by gentamycin assay.

### Differentially Expressed HeLa Cell genes over a Time Course of *M. hominis* Infection

To elucidate the host-reactions at the different stages of *M. hominis* infection, four time points post infection were chosen for microarray gene expression analyses: 0 h to monitor the baseline of transcription, 4 h to examine host reactions to mycoplasma attachment, 48 h to capture in addition the initiation of invasion, and 2 weeks post infection to examine a chronically infected host cell. Starting with a 100-fold multiplicity of infection the cell counts of *M. hominis* and HeLa cells recovered from each time point of infection kept each within the same range (10^5^ – 10^6^ *M. hominis* genome equivalents/PCR and 10^3^–10^4^ HeLa genome equivalents/PCR, see Figure S1). In calculating the ratio of *M. hominis* to HeLa cells a remarkable decrease of mycoplasma cells compared to HeLa cells was detectable from 4 h to 48 h post infection, which might be the result of the host’s effort to eliminate the pathogen. Interestingly, within the chronically infected HeLa cell the ratio of mycoplasma to HeLa cells increased again.

**Figure 2:**
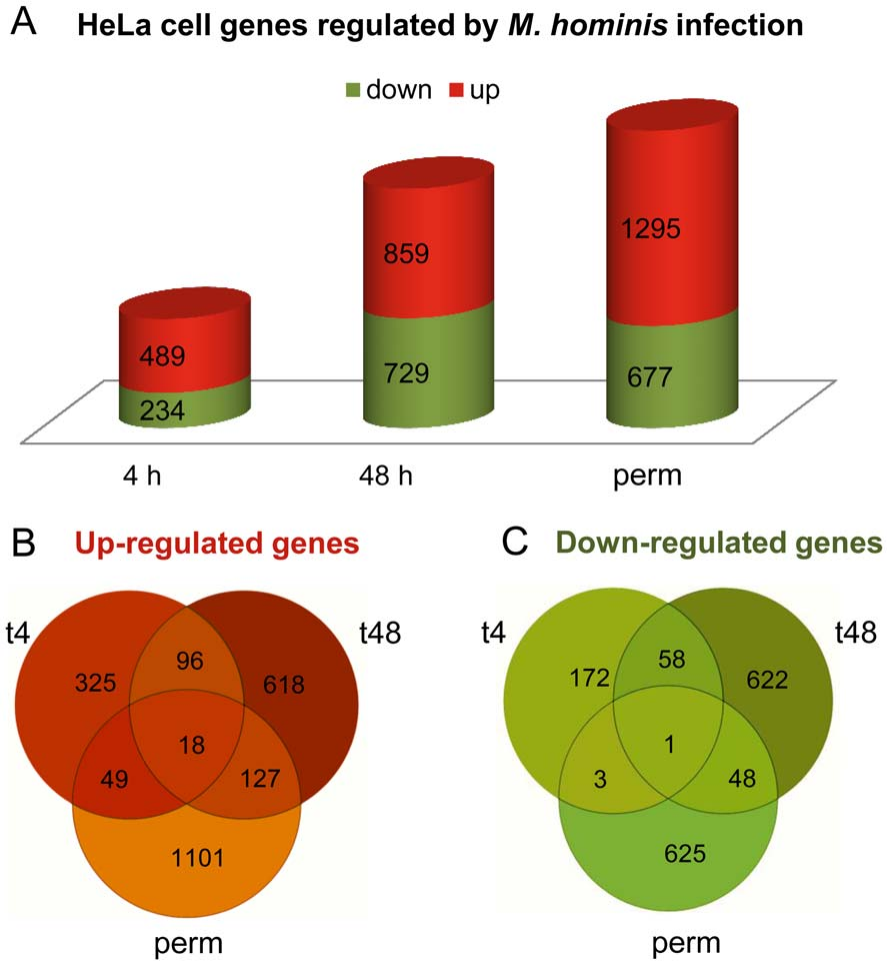
Distribution of differentially regulated HeLa cell genes. Numbers of up- and down-regulated (> 2 fold) HeLa cell genes at 4 h (t4) and 48 h (t48) after infection with *M. hominis* compared to uninfected HeLa cells. A chronically infected HeLa cell line (perm) was compared to HeLa cells infected with *M. hominis* at time point 0 h. A, numbers of up- and down-regulated genes; B, Venn diagram of upregulated genes; C, Venn diagram of down-regulated genes.

Total RNA was prepared from these infection assays in parallel with uninfected HeLa cells and the host cell transcriptomes were determined by comparative microarray analyses. As depicted in Figure 2, a more than twofold change in the expression of 4283 HeLa cell genes was observed over the course of *Mycoplasma hominis* infection compared to the uninfected cell line (all genes, regulated above or below baseline, are listed in Table S1). At 4 h post infection, which corresponds to *M. hominis* attachment and initial colonisation, 67.6% (489/723) of the affected genes were upregulated, whereas at 48 h post infection, when mycoplasma reduction and invasion started, the ratio of up- (859/1588) to down- (729/1588) regulated genes was nearly equal. Almost four times as many genes were exclusively downregulated 48 h after infection and in the chronically infected HeLa cells compared to 4 h post infection, while upregulation of infection-time dependent genes continuously increased from 326 (4 h) to 618 (48 h), to 1101 genes (perm) (see Figure 2, B and C).

### The Most Regulated genes at Each Time Point after Infection

In order to obtain a more detailed understanding of the *Mycoplasma* induced gene expression changes within the host cells we next focused at genes with a more than 10 fold regulation at each time point after infection.

At 4 h post infection 41 genes had a >10 fold difference in expression compared to uninfected cells. Of these 41 genes, 66% (27/41) belonged to one of the three cellular systems: heat shock proteins, the immune system, or cell-cycle regulation. Heat shock protein Hsp70, which plays a role in MAPK signalling and endocytosis pathways, was downregulated in nearly all its subdomain encoding genes (HSPA1A, HSPA1B, HSPA1L and HSPAS2), whereas genes of the immune response, such as the interleukins (IL6, IL11, IL20), their receptors (e.g. IL7R) and several chemokines (CXCL1 and CXCL2), as well as genes involved in cell cycle regulation, cell growth or death (GADD45A, AREG, EREG and metallothioneins), were highly upregulated (Table 1). It should be mentioned that most of the encoded proteins play important roles in more than one system or pathway of the host cell.

**Table 1:** Genes highly up- or downregulated in HeLa cells at different stages of infection with *M. hominis*.

Functions / Pathways	Gene	Fold change / 4 h^a^	Encoded Protein
Heat shock	HSPA1A	−22.40	Heat shock 70kDa protein 1A [NM_005345]
(n = 4)	HSPA1B	−14.84	Heat shock 70kDa protein 1B [NM_005346]
	HSPA1L	−12.27	Heat shock 70kDa protein 1-like [NM_005527]
	HSPA2	−12.25	Heat shock 70kDa protein 2 [NM_021979]
Immune system	IL6	154.51	Interleukin 6 [NM_000600]
(n = 12)	IL11	63.09	Interleukin 11 [NM_000641]
	CCL20	43.54	Chemokine (C-C motif) ligand 20 [NM_004591]
	IL7R	17.33	Interleukin 7 receptor [NM_002185]
	CXCL2	16.82	Chemokine (C-X-C motif) ligand 2 [NM_002089]
	PTGS2	16.50	Prostaglandin-endoperoxide synthase 2 [NM_000963]
	RELB	13.12	V-rel reticuloendothe. viral oncogene homolog B [NM_006509]
	IL1B	13.04	Interleukin 1, beta [NM_000576]
	C1QTNF5	11.51	C1q and tumor necrosis factor related protein 5 [NM_015645]
	IL20	11.31	Interleukin 20[NM_018724]
	CXCL2	10.90	Chemokine (C-X-C motif) ligand 2 [NM_002089]
	MALL	10.85	T-cell differentiation protein-like [NM_005434]
	CXCL1	10.67	Chemokine (C-X-C motif) ligand 1 [NM_001511]
Cell Growth/Death	PKD1L2	48.70	Polycystic kidney disease 1-like 2 [NM_052892]
(n = 11)	MT1F	27.66	Metallothionein 1F [NM_005949]
	CHAC1	25.64	Cation transport regulator homolog 1 [NM_024111]
	MT1X	24.04	Metallothionein 1X (MT1X)[NM_005952]
	CHAC1	22.91	Cation transport regulator homolog 1 [NM_024111]
	MT1M	19.83	Metallothionein 1M [NM_176870]
	PHLDA1	17.61	Pleckstrin homology-like domain [NM_007350]
	SMCR7L	17.07	Smith-Magenis syndr. chrom. region, cand. 7-like [NM_019008]
	EGR1	13.62	Early growth response 1 [NM_001964]
	ERRFI1	11.61	ERBB receptor feedback inhibitor 1 [NM_018948]
	GADD45A	10.17	Growth arrest and DNA-damage-inducible, alpha [NM_001924]
	BIRC3	10.00	Baculoviral IAP repeat-containing 3 [NM_001165]
Signal Transduction	AREG	24.87	Amphiregulin [NM_001657]
(n = 4)	GPR87	23.41	G protein-coupled receptor 87 [NM_023915]
	SH2D1B	18.24	SH2 domain containing 1B [NM_053282]
	EREG	13.24	Epiregulin (EREG)[NM_001432]
Cell Adhesion	AMIGO2	11.21	Adhesion molecule with Ig-like domain 2[NM_181847]
	CLDN1	10.72	Claudin 1 [NM_021101]
Cell Motility	DNAH17	19.01	Heavy chain of dyneine [CR620279]
	ANGPTL4	14.25	Angiopoietin-like 4 [NM_139314]
Others	COLQ	17.10	Collagen-like tail subunit of asymmetric acetylcholinesterase [NM_080538]
	STC2	13.22	Stanniocalcin 2 [NM_003714]
	F3	10.75	Thromboplastin [NM_001993]
	SLC6A7	10.07	Solute carrier family 6 (neurotransmitter transport., L-proline) [NM_014228]
	PADI1	16.07	Peptidyl arginine deiminase, type I [NM_013358]
	RIMS3	−10.64	Regulating synaptic membrane exocytosis 3 [NM_014747]
**Functions / Pathways**	**Gene**	**Fold change / 48 h** a	**Encoded Protein**
Immune System	KLRK1	−22.33	Killer cell lectin-like receptor subfamily K, member 1 [NM_007360]
	IGSF10	−17.58	Immunoglobulin superfamily, member 10 [NM_178822]
	SPINK5	−11.35	Serine peptidase inhibitor, Kazal type 5 [NM_001127698]
	C4BPA	−10.13	Complement component 4 binding protein, alpha [NM_000715]
	IL6	10.52	Interleukin 6 [NM_000600]
Signal Transduction	INSL4	−23.03	Insulin-like 4 (placenta)[NM_002195]
	CACNB2	−13.43	Calcium channel, voltage-dependent, beta 2 subunit [NM_000724]
	FGF21	15.35	Fibroblast growth factor 21 [NM_019113]
	GPR109B	14.78	G protein-coupled receptor 109B [NM_006018]
Cell Growth /Death	LCN2	51.02	Lipocalin 2 [NM_005564]
	MT1M	11.71	Metallothionein 1M [NM_176870]
	CHAC1	11.51	Cation transport regulator homolog 1[NM_024111]
Membrane Proteins	CA9	−11.15	Carbonic anhydrase IX [NM_001216]
	CA12	10.38	Carbonic anhydrase XII [NM_001218]
	SLC7A11	10.30	Solute carrier family 7, member 11 [NM_014331]
ECM-Receptor interaction	SV2B	−14.89	Synaptic vesicle glycoprotein 2B [NM_014848]
	ECM2	12.16	Extracellular matrix protein 2 [NM_001393]
	TIMP4	10.24	TIMP metallopeptidase inhibitor 4 [NM_003256]
Endocrine System	CGA	−23.56	Glycoprotein hormones, alpha polypeptide [NM_000735]
	AGT	11.13	Angiotensinogen (member 8)[NM_000029]
Cell Motility	ANGPTL4	29.00	Angiopoietin-like 4 [NM_139314]
Cell Adhesion	SUSD5	10.11	Sushi domain containing 5[NM_015551]
Development	UNC5B	14.62	Unc-5 homolog B [NM_170744]
Heat shock	HSPA2	−12.52	Heat shock 70kDa protein 2 [NM_021979]
Actin-Cytoskeleton	ACTG2	−13.39	Actin, gamma 2, smooth muscle, enteric [NM_001615]
Lysosome	LAMP3	12.58	Lysosomal-associated membrane protein 3 [NM_014398]
Others	DHRS2	241.69	Dehydrogenase/reductase (SDR family) member 2 [NM_182908]
	KLHDC7B	30.50	Kelch domain containing 7B [NM_138433]
	FAM167A	20.31	Family with sequence similarity 167, member A [NM_053279]
	WDR86	15.21	WD repeat domain 86 [NM_198285]
	METTL10	10.49	Methyltransferase like 10 [NM_212554]
	MAMDC2	−15.32	MAM domain containing 2 [NM_153267]
	ASZ1	−11.17	Ankyrin repeat, SAM and basic leucine zipper domain contain. 1 [NM_130768]
**Functions / Pathways**	**Gene**	**Fold change / perm** a	**Encoded Protein**
Immune system	SAA2	551.74	Serum amyloid A2 [NM_030754]
(n = 22)	S100A8	407.36	S100 calcium binding protein A8 [NM_002964]
	S100A9	89.58	S100 calcium binding protein A9 [NM_002965]
	SAA1	110.14	Serum amyloid A1 [NM_000331]
	C3	79.54	Complement component 3 [NM_000064]
	IL1B	35.86	Interleukin 1, beta [NM_000576]
	CXCL1	33.71	Chemokine (C-X-C motif) ligand 1 [NM_001511]
	TNFAIP6	26.40	Tumor necrosis factor, alpha-induced protein 6 [NM_007115]
	CFB	23.17	Complement factor B [NM_001710]
	PI3	19.41	Peptidase inhibitor 3, skin-derived [NM_002638]
	IL6	15.16	Interleukin 6 (interferon, beta 2)[NM_000600]
	CCL5	13.33	Chemokine (C-C motif) ligand 5 [NM_002985]
	C1S	11.63	Complement component 1, s subcomponent [NM_201442]
	C4BPA	11.31	Complement component 4 binding protein [NM_000715]
	CFH	11.15	Complement factor H [NM_000186]
	IL3RA	11.07	Interleukin 3 receptor, alpha (low affinity) [NM_002183]
	SH2D3C	10.79	SH2 domain containing 3C (SH2D3C)[NM_170600]
	IL24	10.19	Interleukin 24 [NM_006850]
	CXCL2	10.17	Chemokine (C-X-C motif) ligand 2 [NM_002089]
	CCL26	10.05	Chemokine (C-C motif) ligand 26 [NM_006072]
	CHI3L1	10.27	Chitinase 3-like 1 (cartilage glycoprotein-39)[NM_001276]
	C1QTNF5	−10.91	C1q and tumor necrosis factor related protein 5 [NM_015645]
Signal Transduction	BDKRB1	23.86	Bradykinin receptor B1 [NM_000710]
(n = 6)	PTGS2	13.04	Prostaglandin-endoperoxide synthase 2 [NM_000963]
	SPRY4	11.70	Sprouty homolog 4 (Drosophila) [NM_030964]
	AGT	18.98	Angiotensinogen (serpin peptidase inhibitor, member 8) [NM_000029]
	NTS	10.06	Neurotensin [NM_006183]
	GEM	−10.62	GTP binding protein overexpressed in skeletal muscle [NM_005261]
Cell differentiation	LRG1	15.89	Leucine-rich alpha-2-glycoprotein 1 [NM_052972]
(n = 4)	SERPINB3	103.42	Serpin peptidase inhibitor, clade B (ovalbumin) [NM_006919]
	SERPINB4	101.77	Serpin peptidase inhibitor, clade B (ovalbumin) [NM_002974]
	LCN2	62.71	Lipocalin 2 [NM_005564]
Transmembrane Transport	SLC5A10	14.15	Solute carrier family 5 (sodium/glucose cotransporter)[NM_152351]
(n = 4)	SLC39A8	13.12	Solute carrier family 39 (zinc transporter), member 8 [NM_001135147]
	CP	11.22	Ceruloplasmin (ferroxidase) [NM_000096]
	SV2B	−19.21	Synaptic vesicle glycoprotein 2B [NM_014848]
ECM -organisation/-receptor	SPON2	14.55	Spondin 2, extracellular matrix protein [NM_012445]
(n = 3)	MMP1	11.03	Matrix metallopeptidase 1 (interstitial collagenase) [NM_002421]
	ITGB3	12.93	Integrin, beta 3 (platelet glycoprotein IIIa, antigen CD61) [NM_000212]
Ion channel	CACNG6	29.26	Calcium channel, voltage-dependent, gamma subunit 6[NM_145814]
(n = 3)	PKD1L2	−16.28	Polycystic kidney disease 1-like 2 [NM_052892]
	ANO2	−14.17	Anoctamin 2 [NM_020373]
Actin Cytoskeleton	ACTG2	−14.90	Actin, gamma 2, smooth muscle, enteric [NM_001615]
Others	UCA1	297.85	Urothelial cancer associated 1 (non-protein coding) [NR_015379]
(n = 14)	DQX1	20.47	DEAQ box RNA-dependent ATPase 1 [NM_133637]
	EHF	17.51	Ets homologous factor [NM_012153]
	PDZK1IP1	17.21	PDZK1 interacting protein 1[NM_005764]
	FAM65C	15.13	Family with sequence similarity 65, member C [NM_080829]
	CP	14.16	Ceruloplasmin [NM_000096]
	PSCA	13.84	Prostate stem cell antigen [NM_005672]
	AKR1C1	13.43	Aldo-keto reductase family 1, member C1 [NM_001353]
	CYP1A1	12.45	Cytochrome P450, family 1, subfamily A, polypeptide 1 [NM_000499]
	FRMD3	11.13	FERM domain containing 3 [NM_174938]
	SPINK4	10.24	Serine peptidase inhibitor, Kazal type 4 [NM_014471]
	TMEM45B	10.22	Transmembrane protein 45B [NM_138788]
	FAM179A	−19.74	Family with sequence similarity 179, member A [NM_199280]
	SMCR7L	−10.26	Smith-Magenis syndrome chromosome region, cand. 7-like [NM_019008]

a Fold change of upregulated (bold)/ downregulated (-) HeLa cell genes at 4 h, 48 h or 2 weeks (perm) after infection with *M. hominis.*

Eleven of the 12 genes with the greatest differences in expression that did not belong to one of the above mentioned systems were up-regulated, including ANGPTL4. Expression of this gene is known to be upregulated in infection, inflammation or malignancy [15]. The encoded angiopoietin–like 4 protein affects cell-matrix communication [16], interacts with extracellular matrix (ECM) proteins, inhibits proliferation, migration, and tubule formation of endothelial cells [17], and protects endothelial cells from apoptosis [18].

**Figure 3:**
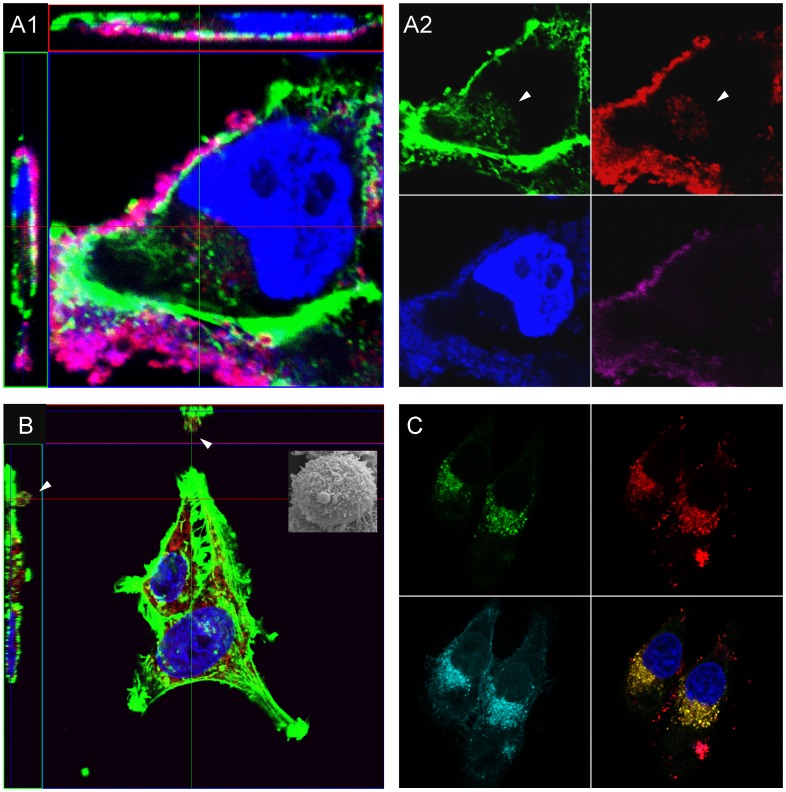
Confocal microscopy of *M. hominis* invasion into, and bleb formation for evasion of HeLa cells. A, HeLa cells analysed at 48 h post infection with *M. hominis* at an MOI of 500 by confocal laser microscopy. Actin filaments were stained green (Alexa 488 phalloidin), nuclei blue (DAPI), surface-attached mycoplasmas magenta (mAb BG11 without fixation) and internalized mycoplasma cells red (mAb BG11 after fixation and permeabilisation). The four-coloured image (A1) is also shown in four single coloured pictures (A2) for a better discrimination of intra- and extra-cellular mycoplasma cells and demonstration of actin rearrangement at the location of intracellular mycoplasmas. B, HeLa cells chronically infected with *M. hominis*. Actin filaments are stained green (Alexa 488 phalloidin), nuclei blue (DAPI), *M. hominis* cells red (mAb BG11). A *M. hominis*-filled protrusion of the HeLa cell membrane bordered by actin (green) is marked by an arrowhead. Inset: SEM of a chronically infected HeLa cell with a prominent protrusion. C, HeLa cells chronically infected with *M. hominis*. LAMP3 proteins are stained green (mAb MEM-259), *M. hominis* cells red (mAb BG11), membranes cyan (Alexa Fluor 594 Wheat Germ Agglutinin) and nuclei blue (DAPI). Co-localisation of LAMP3 and *M. hominis* appears yellow in a three colour overlay (omitting stain of the membranes).

At 48 h post infection 33 genes were differentially expressed more than 10 fold compared to uninfected HeLa cells. In contrast to the host’s reaction at 4 h post infection, the genes differentially regulated at 48 h were associated with a diversity of functions and more than a third (13/33) of these were downregulated. Depending on the multiplicity of infection, a rearrangement of the host’s actin cytoskeleton occurred, resulting in co-localisation with invading mycoplasmas (Fig. 3). The fact that angiopoietin is known to limit the formation of actin stress fibres correlates well with our findings that at 48 h post infection transcription of ANGPTL4 further increased and transcription of gamma actin (ACTG1 and ACTG2) declined. Repression of the heat shock response was less prominent, whereas expression of lysosome-specific markers changed, with upregulation of the lysosome-associated membrane protein LAMP3, more so than LAMP1 and LAMP2, which are delivered to phagosomes during the maturation process. Moreover, expression of the lysosomal cathepsin D (CTSD), which is involved in resolution of inflammation by initiating neutrophil apoptosis [19], increased. Overall, these changes in gene expression correlate well with the reduction of *M. hominis* cells per host cell and demonstrate the host cells’ effort to eliminate the invading pathogen.

The strongest reaction, in terms of gene expression, of the host to *M. hominis* infection was observed in chronically infected cells (2 weeks). Within this chronically infected HeLa cell line, differential transcription was seen in 56 genes (> 10 fold), with 49 upregulated and 7 downregulated. Of these genes 39% (22/56) were functionally related to the immune system, although the genes involved were quite different to those involved at the beginning of the infection. In these chronically infected cells prominent transcription of IL1B, IL24 and IL3RA, all of which influence pathways of MAPK signalling pathways, cytokine-cytokine interactions and apoptosis, was seen. Similarly induced were genes encoding the S100 calcium binding proteins A8 and A9, which are associated with inflammation, antimicrobial activity and apoptosis (reviewed by Goyette & Geczy [20]), and those of the serum amyloid proteins SAA1 and SAA2, which are thought to affect inflammatory responses, to activate TLR2- and TLR4- dependent signalling and to have an intrinsic bactericidal action [21]. Also highly expressed were membrane-embedded proteins, such as the bradykinin receptors B1 and B2, which are involved in signal transduction, and also lipocalin 2 (LCN2), which is associated with pro- as well as anti-apoptotic processes [22].

In intact tissue the extracellular matrix (ECM), which provides structural support for host cells, is covered by epithelial or endothelial cells and is therefore not accessible for bacterial binding and colonisation. However, tissue damage leads to exposure of the ECM, which may facilitate bacterial colonisation [23]. Thus, it is not surprising that in the chronically infected HeLa cells, transcription of genes involved in ECM-receptor interactions was affected. Expression of SV2B, which has been shown to be involved in the regulation of synaptic vesicle exocytosis [24], was more reduced at 48 h post infection than in the early stages of infection (Table 1), whereas expression of the phagosome-related integrin (ITGB3), which functions as an ECM receptor, and the serpins B3 and B4, which bind heparin, leading to inhibition of the lysosomal cysteine protease cathepsin L, was markedly increased.

**Figure 4:**
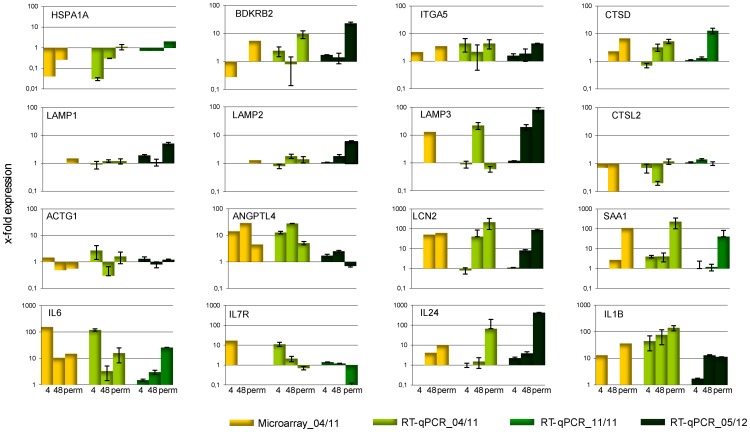
Comparison of microarray and RT-qPCR results. Different preparations of total RNA of HeLa cells (04/11, 11/11 and 05/12) infected with *M. hominis* for 4 h, 48 h or 2 weeks (perm) at an MOI of 100 were subjected to microarray or RT-qPCR analyses and the fold change in expression levels of the named genes, with respect to that in uninfected HeLa cells, quantified as described in the Method section.

To verify the microarray results, transcript levels of specific genes were confirmed by RT-qPCR using RNA purified from the same infection assays used for the microarrays and, additionally, RNA from two separate infection assays conducted months later. As shown in Figure 4, some differences became obvious when using RNA from independent infection assays. In particular some of the most highly regulated genes (e.g. IL6) did not exhibit the same expression profiles. Expression levels at 4 h and 48 h and in the chronically infected HeLa cells (perm) measured by microarray corresponded with those quantified by RT-qPCR when using the same RNA preparation. The differences are more likely to result from slightly different infection conditions, than of inaccuracies in measuring expression levels by microarray, and may be an indicator of variation in the capacity of the host cell to modulate the transcriptional response to the infectious pathogen used.

### *M. hominis* Infection Affected HeLa Cell Pathways

As the quantification of the expression levels of distinct genes does not necessarily provide a complete picture of what is happening in the host cell at different stages of infection, and many regulated factors are known to function at several sites in the cell, sometimes fulfilling different functions in different locations, we looked at which functional systems, networks or pathways the more regulated genes clustered. We mapped the differentially expressed transcripts derived from our microarray analyses onto KEGG pathway maps [25], which represent current knowledge of molecular interactions and reaction network systems for Metabolism (1), Genetic Information Processing (2), Environmental Information Processing (3), Cellular Processes (4) and Organismal Systems (5). We also calculated the ratio of regulated genes per total number of genes in each pathway (Table 2).

**Table 2:** Number of pathway-assigned HeLa cell genes regulated at the different stages of infection with *M. hominis*.

	*Number of genes*			
*Pathways*	*Total*	*Regulated at 4 h* a	*Regulated at 48 h* a	*Regulated in chronic infection* a
01100 Metabolic pathways	1123	37 (3.3%)	101 (9.0%)	97 (8.6%)
**Transcripts regulated in Metabolism**	**1123**	**37 (3.3%)**	**101 (9.0%)**	**97 (8.6%)**
Transcription				
03020 RNA polymerase	30	–	1 (3.3%)	-
03022 Basal transcription factors	43	–	1 (2.3%)	-
03040 Spliceosome	127	5 (3.9%)	7 (5.5%)	2 (1.6%)
Translation				
03010 Ribosome	88	3 (3.4%)	5 (5.7%)	2 (2.3%)
03013 RNA transport	153	1 (0.7%)	4 (2.6%)	5 (3.3%)
03015 mRNA surveillance pathway	89	–	2 (2.2%)	3 (3.4%)
03008 Ribosome biogenesis in eukaryotes	75	–	3 (4.0 %)	3 (4.0%)
Folding, Sorting and Degradation				
03060 Protein export	23	–	–	1 (4.3%)
04141 Protein processing in endoplasmic reticulum	166	10 (6.0%)	16 (9.6%)	8 (4.8%)
04130 SNARE interactions in vesicular transport	36	1 (2.8%)	2 (5.6%)	1 (2.8%)
04120 Ubiquitin mediated proteolysis	137	4 (2.9%)	5 (3.6%)	9 (6.6%)
03050 Proteasome	44	–	1 (2.3%)	–
03018 RNA degradation	71	2 (2.8%)	4 (5.6%)	1 (1.4%)
Replication and Repair				
03030 DNA replication	36	–	1 (2.8%)	2 (5.6%)
03410 Base excision repair	32	–	***4 (12.5%)***	1 (3.1%)
03420 Nucleotide excision repair	44	–	2 (4.5%)	2 (4.5%)
03430 Mismatch repair	23	–	–	1 (4.3%)
03440 Homologous recombination	28	–	1 (3.6%)	1 (3.6%)
03450 Non-homologous end-joining	13	–	1 (7.7%)	–
**Transcripts regulated in Genetic Information Processing**	**1258**	**26 (2.1%)**	**60 (4.8%)**	**42 (3.3%)**
Membrane Transport				
02010 ABC transporters	44	–	3 (6.8%)	***5 (11.4%)***
Signal Transduction				
04010 MAPK signaling pathway	267	26 (9.7%)	23 (8.6%)	26 (9.7%)
04012 ErbB signaling pathway	87	***10 (11.5%)***	4 (4.6%)	8 (9.2%)
04310 Wnt signaling pathway	151	10 (6.6%)	9 (6.0%)	13 (8.6%)
04330 Notch signaling pathway	47	3 (6.4%)	***6 (12.8%)***	3 (6.4%)
04340 Hedgehog signaling pathway	56	5 (8.9%)	4 (7.1%)	5 (8.9%)
04350 TGF-beta signaling pathway	84	***9 (10.7%)***	***9 (10.7%)***	***12 (14.3%)***
04370 VEGF signaling pathway	75	2 (2.7%)	***8 (10.7%)***	7 (9.3%)
04630 Jak-STAT signaling pathway	129	***16 (12.4%)***	7 (5.4%)	***13 (10.1%)***
04020 Calcium signaling pathway	177	8 (4.5%)	14 (7.9%)	13 (7.3%)
04070 Phosphatidylinositol signaling system	80	–	***9 (11.3%)***	6 (7.5%)
04150 mTOR signaling pathway	52	3 (5.8%)	4 (7.7%)	2 (3.8%)
Signalling Molecules and Interaction				
04080 Neuroactive ligand-receptor interaction	273	7 (2.6%)	11 (4.0%)	18 (6.6%)
04060 Cytokine-cytokine receptor interaction	258	***27 (10.5%)***	20 (7.8%)	***27 (10.5%)***
04512 ECM-receptor interaction	84	8 (9.5%)	4 (4.8%)	***11 (13.1%)***
04514 Cell adhesion molecules (CAMs)	132	5 (3.8%)	3 (2.3%)	7 (5.3%)
**Transcripts regulated in Environmental Information Processing**	**1996**	**139 (7.0%)**	**138 (6.9%)**	**176 (8.8%)**
Transport and Catabolism				
04144 Endocytosis	202	14 (6.9%)	19 (9.4%)	***23 (11.4%)***
04145 Phagosome	147	5 (3.4%)	7 (4.8%)	14 (9.5%)
04142 Lysosome	120	2 (1.7%)	***17 (14.2%)***	***22 (18.3%)***
04146 Peroxisome	78	–	***11 (14.1%)***	***8 (10.3%)***
04140 Regulation of autophagy	30	2 (6.7%)	1 (3.3%)	1 (3.3%)
Cell Motility				
04810 Regulation of actin cytoskeleton	212	11 (5.2%)	18 (8.5%)	19 (9.0%)
Cell Growth and Death				
04110 Cell cycle	124	12 (9.7%)	9 (7.3%)	5 (4.0%)
04114 Oocyte meiosis	112	2 (1.8%)	9 (8.0%)	5 (4.5%)
04210 Apoptosis	87	***9 (10.3%)***	6 (6.9%)	***9 (10.3%)***
04115 p53 signaling pathway	68	***13 (19.1%)***	***13 (19.1%)***	***7 (10.3%)***
Cell Communication				
04510 Focal adhesion	200	19 (9.5%)	16 (8.0%)	***21 (10.5%)***
04520 Adherens junction	73	5 (6.8%)	5 (6.8%)	5 (6.8%)
04530 Tight junction	131	1 (0.8%)	9 (6.9%)	7 (5.3%)
04540 Gap junction	88	–	3 (3.4%)	***10 (11.4%)***
**Transcripts regulated in Cellular Processes**	**1672**	**95 (5.7%)**	**143 (8.6%)**	**156 (9.3%)**
Immune System				
04640 Hematopoietic cell lineage	86	8 (9.3%)	6 (7.0%)	***9 (10.5%)***
04610 Complement and coagulation cascades	68	***8 (11.8%)***	***10 (14.7%)***	***13 (19.1%)***
04620 Toll-like receptor signaling	98	7 (7.1%)	6 (6.1%)	***12 (12.2%)***
04621 NOD-like receptor signaling	57	10 (17.5%)	5 (8.8%)	***12 (21.1%)***
04622 RIG-I-like receptor signaling	67	3 (4.5%)	3 (4.5%)	***7 (10.4%)***
04623 Cytosolic DNA-sensing pathway	57	4 (7.0%)	4 (7.0%)	***6 (10.5%)***
04650 Natural killer cell mediated cytotoxicity	124	5 (4.0%)	12 (9.7%)	11 (8.9%)
04612 Antigen processing and presentation	71	6 (8.5%)	6 (8.5%)	2 (2.8%)
04660 T cell receptor signaling pathway	108	10 (9.3%)	5 (4.6%)	8 (7.4%)
04662 B cell receptor signaling pathway	76	6 (7.9%)	7 (9.2%)	6 (7.9%)
04664 Fc epsilon RI signaling pathway	78	3 (3.8%)	5 (6.4%)	7 (9.0%)
04666 Fc gamma R-mediated phagocytosis	93	4 (4.3%)	8 (8.6%)	8 (8.6%)
04670 Leukocyte transendothelial migration	115	3 (2.6%)	10 (8.7%)	11 (9.6%)
04672 Intestinal immune network for IgA production	48	2 (4.2%)	3 (6.3%)	3 (6.3%)
04062 Chemokine signaling pathway	186	7 (3.8%)	9 (4.8%)	18 (9.7%)
Endocrine System				
04910 Insulin signaling pathway	138	10 (7.2%)	***17 (12.3%)***	8 (5.8%)
04920 Adipocytokine signaling pathway	69	***7 (10.1%)***	***8 (11.6%)***	6 (8.7%)
03320 PPAR signaling pathway	71	4 (5.6%)	***10 (14.1%)***	***8 (11.3%)***
04912 GnRH signaling pathway	100	4 (4.0%)	8 (8.0%)	***10 (10.0%)***
04914 Progesterone-mediated oocyte maturation	86	2 (2.3%)	5 (5.8%)	3 (3.5%)
04916 Melanogenesis	101	4 (4.0%)	10 (9.9%)	***11 (10.9%)***
04614 Renin-angiotensin system	17	–	***3 (17.6%)***	***2 (11.8%)***
Circulatory System				
04260 Cardiac muscle contraction	72	1 (1.4%)	5 (6.9%)	6 (8.3%)
04270 Vascular smooth muscle contraction	115	3 (2.6%)	15 (13.0%)	***12 (10.4%)***
Digestive System				
04970 Salivary secretion	84	1 (1.2%)	***10 (11.9%)***	8 (9.5%)
04971 Gastric acid secretion	74	2 (2.7%)	***8 (10.8%)***	5 (6.8%)
04972 Pancreatic secretion	98	–	6 (6.1%)	9 (9.2%)
04976 Bile secretion	71	1 (1.4%)	7 (9.9%)	6 (8.5%)
04973 Carbohydrate digestion and absorption	40	1 (2.5%)	***6 (15.0%)***	2 (5.0%)
04974 Protein digestion and absorption	79	2 (2.5%)	7 (8.9%)	3 (3.8%)
04975 Fat digestion and absorption	45	–	2 (4.4%)	2 (4.4%)
04977 Vitamin digestion and absorption	24	–	1 (4.2%)	–
04978 Mineral absorption	51	***9 (17.6%)***	***7 (13.7%)***	***10 (19.6%)***
Excretory System				
04962 Vasopressin-regulated water reabsorption	44	2 (4.5%)	***5 (11.4%)***	2 (4.5%)
04960 Aldosterone-regulated sodium reabsorption	41	–	***5 (12.2%)***	3 (7.3%)
04961 Endocrine and other factor- regulated calcium reabsorption	49	1 (2.0%)	***5 (10.2%)***	7 (14.3%)
04964 Proximal tubule bicarbonate reclamation	23	1 (4.3%)	***3 (13.0%)***	1 (4.3%)
04966 Collecting duct acid secretion	26	–	1 (3.8%)	1 (3.8%)
Nervous System				
04724 Glutamatergic synapse	125	1 (0.8%)	8 (6.4%)	9 (7.2%)
04725 Cholinergic synapse	112	2 (1.8%)	9 (8.0%)	9 (8.0%)
04720 Long-term potentiation	70	–	6 (8.6%)	5 (7.1%)
04730 Long-term depression	69	1 (1.4%)	4 (5.8%)	***8 (11.6%)***
04721 Synaptic vesicle cycle	63	3 (4.8%)	6 (9.5%)	3 (4.8%)
04722 Neurotrophin signaling pathway	127	5 (3.9%)	5 (3.9%)	10 (7.9%)
Sensory System				
04740 Olfactory transduction	387	1 (0.3%)	3 (0.8%)	1 (0.3%)
04742 Taste transduction	52	–	–	2 (3.8%)
Development				
04320 Dorso-ventral axis formation	24	2 (8.3%)	***3 (12.5%)***	2 (8.3%)
04360 Axon guidance	129	5 (3.9%)	7 (5.4%)	9 (7.0%)
04380 Osteoclast differentiation	126	12 (9.5%)	8 (6.3%)	***17 (13.5%)***
Environmental Adaptation				
04710 Circadian rhythm - mammal	22	2 (9.1%)	2 (9.1%)	1 (4.5%)
***Transcripts regulated in Organismal Systems***	***4156***	***175 (4.2%)***	***314 (7.6%)***	***334 (8.0%)***

a number (and percentage) of pathway genes that are up- or downregulated for more than twofold; cells in which more than 10% of the pathway genes are regulated are shown in bold and italics.

**Figure 5:**
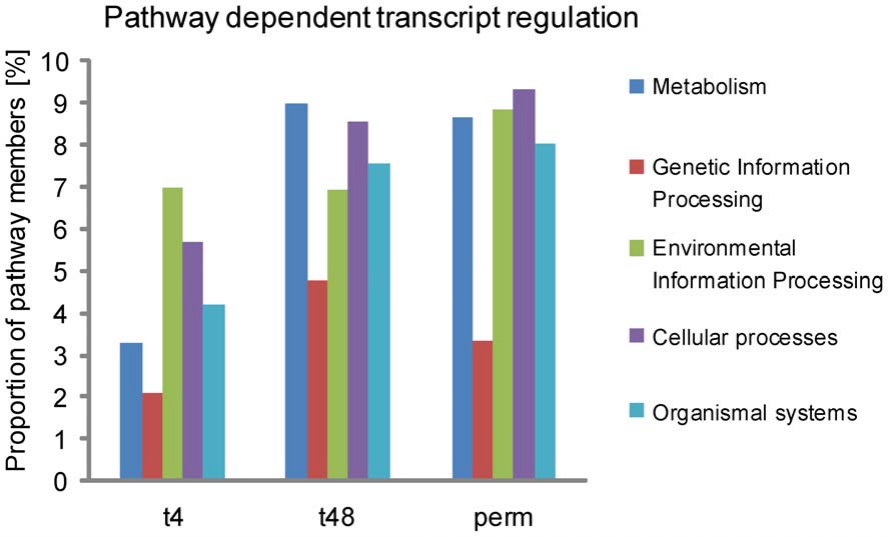
Network systems’ dependent transcript regulation. Regulated HeLa cell genes at 4 h (t4), 48 h (t48) and 2 weeks (perm) after infection with *M. hominis* were sorted to KEGG- pathway maps and integrated into those belonging to the Metabolism, Genetic or Environmental Information Processing, Cellular Processes and Organismal Systems groups. The proportions of pathway genes that were regulated are shown.

At all stages of infection, Genetic Information Processing (such as *transcription*, *translation*, *folding, sorting and degradation,* and *replication and repair*) was the least affected system of the host (Fig. 5). This correlates well with previous observations of reduced growth in *M. hominis*-infected HeLa cells [6]. At an early stage of infection (4 h), the regulated genes were found predominantly in pathways belonging to Environmental Information Processing (7% of which were regulated), such as *signal transduction*, and *signalling molecules and interaction.* In the second most regulated system, Cellular Processes, 5.7% of the genes were differentially expressed, and in Organismal Systems 4.2% of the genes were regulated (Fig. 5, 4 h). Metabolic pathways were mainly affected at later stages of infection (9% of the genes being differentially regulated at 48 h post infection and 8.6% in the chronically infected cells). With the exception of Genetic Information Processing, more regulated genes were found in pathways in the chronically infected HeLa cell, demonstrating the host’s ongoing struggle against the pathogen (Fig. 5, perm).

### Identification of Regulated Pathways in Environmental Information Processing

As might be expected as part of the host reaction to attachment of mycoplasmas 4 h post infection, most of the differentially expressed genes (constituting more than 10% of the genes in the pathway) occurred in *signal transduction*. In the Jak-STAT signalling pathway 12.4% of the genes were differentially expressed at the beginning of infection, including greatly increased expression (>10 fold) of IL6, IL7R, IL11 and IL20. The number of regulated genes declined at 48 h post infection to 5.4% and increased again in the chronically infected cells to constitute 10.1% of the genes in this category, with a modified spectrum of highly induced genes, namely IL3RA, IL6, IL24 and SPRY4.

At 48 h post infection signal transduction was spread over several signalling pathways. More than 10% of the genes in each of the following signalling pathways were differentially expressed: the Notch signalling pathway (hsa04330), the TGF-beta signalling pathway (hsa4350), the VEGF signalling pathway (hsa04370) and the phosphatidylinositol signalling system (hsa04070). Regulation of genes’ expression involved in ECM-receptor interactions was highest in the chronically infected HeLa cells, in which 13.1% of the pathway genes were differentially expressed including SV2B, which was highly downregulated and the integrins which were highly upregulated in number (n = 6) and concentration (ITGB3 = 13 fold).

A high proportion of highly upregulated genes was also found in the cytokine-cytokine receptor interaction pathway (hsa04060), which belongs to the *signalling molecules and interaction* group of the Environmental Information Processing system, with 27 of the 258 pathway genes (10.5%) upregulated 4 hours post infection, eight of them, encoding IL1B, IL6, IL11, IL7R, IL20, CCL20; CXCL1 and CXCL2, by more than tenfold. Although the proportion of regulated transcripts remained constant in the chronically infected cell line, the pattern of highly expressed cytokines (>10 fold) changed; IL11, IL7R, IL20, CCl20 and CXCL2 decreased, and CCL5, CCL26, IL3RA and IL24, which increased in expression.

### Identification of Regulated Pathways in Cellular Processes and Organismal Systems

The total proportion of regulated transcripts in Cellular Processes was 5.7% (95/1672) at 4 h post infection with 19.1% (13/68) of those genes being concentrated in the p53 signalling pathway (hsa04115), demonstrating the importance of this pathway in early host responses to the attached pathogen. In the apoptotic pathway (hsa04210) 10.3% of the genes (9/87) were regulated, with BIRC3 and IL1B more than 10-fold upregulated.

Differentially expressed genes in the Organismal System network clustered in two pathways of the *immune system* group, those for NOD-like receptor signalling and the complement cascade. In the NOD-like receptor signalling pathway (hsa04621) 10 genes (of a total of 57 pathway genes) were differentially expressed at 4 h post infection and five of them were upregulated more than 10-fold, encoding IL6, IL1B, BIRC3, CXCL1 and CXCL2. The NOD-like receptor signalling pathway seemed to represent an important route for host cell identification of *M. hominis* as a pathogen and for the generation of an innate immune response. This was supported by the findings that in the chronically infected HeLa cells the proportion of regulated genes in this cluster increased to 21.1% (12/57), with little change in the highly induced genes encoding IL1B, IL6, CCL5 and CXCL1.

The complement and coagulation cascades (hsa04610) were mainly affected in the chronically infected HeLa cells, with 19.1% of the pathway genes differentially expressed compared to the uninfected host cells, with five genes highly upregulated. These encode the complement factors C4BPA, a component of the classical pathway, CFB and CFH, components of the alternative pathway, C3, a component of both complement activation pathways, and BDKRB1, which, in healthy human tissues, is rarely expressed at significant levels [26].

## Discussion

**Figure 6:**
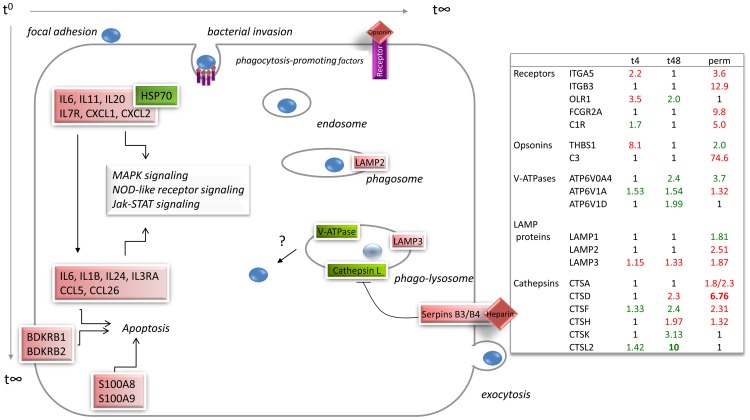
Model of *M. hominis* infection of HeLa cells. Based on the chronology of the host response to a *M. hominis* infection, a model of infection-stage dependent colonization with *M. hominis* cell (blue sphere) was developed. Infection begins at t^0^ with focal adhesion, followed by opsonin-receptor-mediated phagocytosis and different stages of phago-lysosome maturation, ending at t∞, either with a protrusion of the host cell membrane as a prerequisite for mycoplasmal exocytosis or with a postulated escape into the cytoplasm of the host cell (?) or in destruction in the lysosome (light blue sphere). Infection-stage dependent genes of the immune system are upregulated, affecting Jak-STAT, MAPK and NOD-like receptor signalling and apoptosis. Upregulated genes are coloured light-red and down-regulated genes light-green. Fold changes in expression of genes were calculated from microarray results and represents the mean of quadruplicate assays.

To study the host‘s reactions to infection with *M. hominis* in an *in vitro* model, we performed scanning electron microscopy, confocal microscopy and transcriptome analyses of HeLa cells infected with *M. hominis* strain FBG for 4 h, 48 h and two weeks. We were able to study the host reaction to infection with a bacterium harbouring only 537 annotated proteins (the second smallest known mycoplasmal genome). Infection-stage dependent changes in the host cell were elucidated by mapping the regulated genes to the KEGG Pathways and developing a *M. hominis*-specific infection model (Fig. 6). This goal was complicated by the fact that at the different time points post infection, mixtures of HeLa cells were analysed that were either uninfected, or attached by, or attached and invaded by *M. hominis*. Furthermore the up- and downstream neighbours of a highly regulated signalling pathway component were often not regulated, which may be due to the different half-lives of transcripts or, alternatively, unknown regulatory mechanisms. Differential expression of genes was confirmed by RT-qPCR and correlated well when using the same RNA, but differed in some cases when using RNA isolated from different infection assays at different times, possibly demonstrating the variability of individual host-pathogen interactions.

As depicted in Fig. 6, mycoplasmal infection of HeLa cells starts with cytoadhesion which has previously been shown to be mediated by cytoadhesins such as P50/Vaa, P60/P80 and OppA [2]. A prominent induction of pro- and anti-apoptotic cytokines occurs and HSP70 is highly repressed. Besides their action as molecular chaperones, heat shock proteins are known to modulate the innate and adaptive immune responses after exposure to microbial pathogens. Therefore, downregulation of HSP70 has been interpreted as a mechanism of immune evasion and it has been suggested that this promotes chronic infection [27]. This is in accordance with observations that the course of a *M. hominis* infection is rarely acute, and that the *M. hominis* induced cytokine profile in human dendritic cells corresponds to that of a pathogen associated with chronic infections [28].

Targosz and coworkers demonstrated in 2006 that *Helicobacter pylori* also inhibits expression of HSP70 in human epithelial cells [29]. They hypothesised that downregulation of HSP70 might be a primary cause of Helicobacter-associated mucosal cell damage. The data of Axsen and coworkers confirmed a reduced HSP expression in *H. pylori*-infected cell lines and in animal models, which was interpreted as a mechanism of immune evasion [30]. In 2009 Kang et al. showed that downregulation of HSP70 protects rheumatoid arthritis fibroblast-like synoviocytes from NO-induced apoptosis [31]. Interestingly, *M. hominis* is known to be associated with arthritis [32] with symptoms similar to rheumatoid arthritis [33] known to be the result of an autoantibody response.

Cytoadhesins of *M. hominis* have been shown to bind to sulphated structures on human cells and ECM molecules [3], [34]. This is in good accordance with the upregulation of laminin, thrombospondin and collagen as ligands, and integrins as receptors in the host cell membrane for internalisation (Table S1). *M. hominis* neither possesses a type III secretion system like *Salmonella* or *Shigella* (which enables uptake according to the trigger model) nor induces e-cadherin nor prominent MET expression, as shown for *Listeria* invasion (which follows the zipper model) [35]. Nevertheless, confocal microscopy analyses in this study clearly demonstrated that *M. hominis* invades the HeLa cell and that, depending on the multiplicity of infection, this is associated with rearrangement of the actin cytoskeleton (see Fig. 3.A).

Invasive pathogens have evolved efficient strategies to promote their uptake in non-phagocytic cells such as HeLa cells. To do so they mimic or bind endogenous ligands of host cell receptors, thus activating signalling cascades for uptake. Phagocytosis promoting factors and receptors were found to be upregulated at an early stage of *M. hominis* infection and in chronically infected HeLa cells (Fig. 6). Interestingly, the S100 proteins A8 and A9, the serpins B3 and B4 and bradykinin receptors B1 and B2 were highly upregulated only within the chronically infected HeLa cells. In endothelial cells the human S100 A8/A9 heterocomplex has been shown to induce expression of adhesion molecules and a weak increase in pro-inflammatory cytokines [36], in accordance with our findings that integrins and a distinct set of cytokines, including IL-1 beta (IL1B), were upregulated. The complexity of the network is evident from the observation that IL-1 beta upregulates the expression of the bradykinin receptors B1 and B2 [37]. In infection with Listeria, immunity to the bacterium has been shown to be via activation of the BDKRB2 receptor [38] and in infections with *Staphylococcus aureus* or *Pseudomonas aeruginosa*, secreted components of the pathogens have been demonstrated to upregulate human BDKRB1 in epithelial cells via NFkB-pathways [39], [40]. Thus, upregulation of the bradykinin receptors B1 and B2 seems to be an important host defense mechanism against invading microbes including *M. hominis*.

In *Listeria* infection, serpins and clathrin have been shown to be mediators of bacterial endocytosis rather than phagocytosis [41]. As clathrin was moderately down-regulated in *M. hominis*-infected HeLa cells, activation of serpins B3 and B4 seems more likely to be associated with the phagocytotic process. Both serpins have been shown to inhibit the cysteine protease cathepsin L, which is normally highly expressed in lysosomes [42] and, indeed, in *M. hominis*-infected HeLa cells, cathepsin L2 is downregulated 48 h post infection. Viable *Salmonella* Typhimurium are found in special phagosomes of macrophages that diverge from the “normal” degradative endocytic pathway in lacking cathepsin L2 and neither interact with incoming endocytic components such as transferrin and dextran nor fuse with functional lysosomes [43], thus preventing their elimination.

Once within the host cell, bacteria have evolved different mechanisms for survival. *Legionella pneumophila*, *Brucella abortus* and *Porphyromonas gingivalis* invade endothelial cells and replicate in vacuoles that resemble autophagosomes. Here the late endosomal markers and lysosomal cathepsin L are absent [44]. The late endosomal marker LAMP3 (lysosome-associated marker protein 3) was markedly upregulated in HeLa cells chronically infected with *M. hominis*. As viable, proliferating *Pseudomonas aeruginosa* are only found in perinuclear vacuoles that do not contain LAMP3 [45], upregulation of LAMP3 may either be indicative of *M. hominis* residence in a lytic surrounding or a prerequisite for host lysosomal clearance of the mycoplasma. Interestingly, it could be demonstrated in confocal microscopy that the invaded *M. hominis* cells co-localise with LAMP3 in the chronically infected HeLa cell line (Fig. 3. C). Thus, a survival in this vacuole becomes most likely and is supported by the findings that an additional acidification of the lysosome by V-ATPases, which is known to affect a pathogen’s clearance, probably does not take place. Transcripts of the lysosomal V-ATPases are nearly all downregulated in the chronically infected HeLa cells (see Fig. 6). In addition, the few upregulated V-ATPase genes reach transcript levels of only up to 1.3 fold higher than in the uninfected HeLa cells. It would be of great interest to examine whether resistance in or escape from the late phago(lyso)some into the cytoplasm is successful and which mycoplasmal effectors are involved. *Listeria monocytogenes* escapes from the phagosome into the cytosol of the invaded cell, where it replicates and, by the use of an actin-based motility mechanism, spreads from cell to cell [46]. The strategy used by the internalized *M. hominis* cell to survive within the HeLa cell can be better understood by examining the published data about its symbiotic relationship with *Trichomonas vaginalis* and our data on the regulated genes of the phagosomal and lysosomal pathways. Vancini and Benchimol published a study in 2008 describing the entry and intracellular localisation of *M. hominis* in *Trichomonas vaginalis* [47]. Furthermore, using transmission electron microscopy, they were able to visualize endocytotic uptake involving coated pits. Depending on the *Trichomonas* strain used, the internalized mycoplasmas were digested, or were able to survive or even to escape from the vacuole into the cytoplasm. One could argue that this endosymbiotic situation is not compatible with a host-pathogen interaction in which an ongoing battle for survival occurs. However, as Brunell et al. reported in 2002, *S.* Typhimurium replicates in vacuoles of non-phagocytic epithelial cells and phagocytic macrophages [48] even though only the latter affects bacterial clearance. Budding of the trichomonal plasma membrane bearing *Mycoplasma hominis* [47] looks similar to the budding observed in this study by confocal laser microscopy (Fig. 3 B) and scanning electron microscopy (inset in 3 B) in chronically infected HeLa cells, suggesting that *M. hominis* could exit HeLa cells in the same way as it leaves *Trichomonas*.

The present study provides an initial overview of the reactions of the *M. hominis*-infected HeLa cell at different time points of infection. The findings suggest a complex, infection-stage dependent intervention of the host directed at mycoplasmal clearance and counteracted by the nature of this pestering pathogen. In the future, studies will be required to confirm whether our model of *M. hominis* infection in HeLa cells is fully representative. The host reactions to intracellular mycoplasmas will need to be characterized separately from the membrane adhesive mycoplasmas (e.g. in eliminating the surface colonising mycoplasmas via gentamycin treatment), the components of *M. hominis*-containing vacuoles will need to be characterized in more detail, and the proposed mycoplasmal escape into the cytoplasm of HeLa cells and the presence of *M. hominis* within these budding-like structures will need to be proven. By characterizing the mycoplasmal effectors that are involved, we will deepen our understanding of the survival strategies of this facultative human pathogen.

## Materials and Methods

### Cell Culture Conditions

The human cervical carcinoma cell line HeLa S3 (ATCC CCL2.2) was cultivated in DMEM medium supplemented with 10% (v/v) horse serum and 0.01% (w/v) penicillin/streptomycin in a humidified 10% CO_2_-enriched atmosphere at 37°C. Cells were harvested by trypsinisation, suspended in 10 ml buffered saline (PBS) and pelleted by subsequent low speed centrifugation (1,100×g, 10 min). The proportion of damaged cells was ascertained using trypan blue staining. The HeLa cell line chronically infected with *M. hominis* was cultivated in the same culture medium. *M. hominis* strain FBG was grown in arginine-medium as described previously [2].

### Infection Assay

Two days pre-infection 30 ml of a mid-logarithmic-phase broth culture of *M. hominis* was suspended in 300 ml DMEM-medium supplemented with 10% (v/v) horse serum, 0.01% (w/v) penicillin/streptomycin and 75 ml arginine-medium and cultivated for 48 h at 37°C. After sedimentation of the mycoplasma cells (4,000×g, 30 min) the sediment was washed with PBS, suspended in 300 ml DMEM/10% (v/v) horse serum/0.01% (w/v) penicillin/ streptomycin; and 10 ml *M. hominis* cell suspension was added to 1×10^7^ HeLa cells that had been seeded 20 h pre-infection in 75 cm^2^ cell culture flasks. For scanning and confocal laser microscopy, HeLa cells were seeded in 24 well tissue culture plates on glass cover slips at 1–2×10^5^cells/well and after 5–48 h of cultivation the cells were infected with 1–2×10^7^ *M. hominis* cells. The multiplicity of infection (MOI) was controlled by measurement of the number of colour changing units (CCU) of *M. hominis*, as previously described [49]. In general, the procedure described above resulted in an MOI of 100. Recovery rates of *M. hominis* and HeLa cells at each time point of infection were estimated by TaqMan based quantification of HeLa (h*gap*) and *M. hominis* (*hit*A) genome equivalents [50], enabling the calculation of cell counts and the respective cell ratios.

At 0 h, 4 h, 24 h and 48 h post infection, *M. hominis*-infected HeLa cells were examined by confocal microscopy or scanning electron microscopy, or subjected to RNA purification for microarray-based transcriptome analyses. At each time point of infection, as well as for the chronically infected HeLa cell line, uninfected HeLa cells were used as negative controls and all samples were analysed in quadruplicate.

### Nucleic Acid Preparations and Gene Expression Analyses

Nucleic acids were prepared from each 75 cm^2^ cell culture flask after twice washing the adherent HeLa cells with 10 ml PBS and subsequent lysis in 650 µl RLT Buffer (RNeasy Kit; Qiagen GmbH, Hilden, Germany). Genomic DNA of each sample was isolated using 50 µl lysate/RTL Buffer adjusted to 200µl with G2-Buffer of the EZ1 DNA Tissue Kit followed by purification on an EZ1 Biorobot after proteinase K digestion according to the instructions of the manufacturer (Qiagen). 600µl lysate/RTL Buffer was used for total RNA preparation according to the instructions of the manufacturer. All RNA samples that were analysed by RT-PCR were pretreated with DNaseI to digest contaminating traces of DNA, as described previously [5]. RNA integrity was checked using an Agilent 2100 Bioanalyzer (Agilent Technologies, Waldbronn, Germany). All samples in this study had high quality RNA integrity numbers (RIN 9.7–10). RNA was quantified photometrically using a Nanodrop ND1000 (Thermo Fisher Scientific GmbH, Dreieich, Germany).

Synthesis of cDNA and subsequent fluorescent labelling of cRNA was performed on four replicates of each experimental condition according to the manufactureŕs protocol (One-Color Microarray-Based Gene Expression Analysis / Low Input Quick Amp Labeling; Agilent Technologies). Briefly, 100 ng of total RNA was converted to cDNA, followed by *in vitro* transcription and incorporation of Cy3-CTP into the nascent cRNA. After fragmentation, labelled cRNA was hybridized to Agilent SurePrint G3 Human GE 8×60k Microarrays (Agilent Technologies, Boeblingen, Germany) for 17 h at 65 °C and scanned as described in the manufactureŕs protocol.

Signal intensities on 20 bit tiff images were calculated using Feature Extraction (FE, Vers. 10.7.1.1; Agilent Technologies). Data analyses were conducted with GeneSpring GX (Vers. 11.5; Agilent Technologies). Probe signal intensities were quantile normalized across all samples to reduce inter-array variability [51]. Input data pre-processing was concluded by baseline transformation to the median of all samples.

After grouping of replicates according to their respective experimental condition a given transcript had to be expressed above background (called “detected” by FE) in at least three of four replicates in any one of two or both conditions to be further analysed in pairwise comparisons of conditions (t4 infected vs. uninfected, t48 infected vs. uninfected, perm infected vs. t0 infected). Differential gene expression was assessed statistically using unpaired T-tests. Resulting P values were corrected for multiple testing [52]. Figure 2 shows the distribution of differentially regulated HeLa cell genes (> 2 fold), while more detailed analyses are shown in Table S1.

### Pathway Analyses

Primary pathway analysis was performed online using the Kyoto Encyclopedia of Genes and Genomes (KEGG) website (http://www.genome.jp/kegg/pathway.html; vers. 7/21/2011). Entrez GeneIDs (including aliases) of differentially expressed genes were searched against human reference pathways within the KEGG basic pathway mapping tool. HTML output was extracted and integrated into a local database for detailed enrichment analyses of regulated genes.

### RT-qPCR

Oligonucleotides were designed using Probefinder (Roche Applied Science) (https://qpcr.probefinder.com).

RNA (1 µg) was converted to random-primed cDNA in a total volume of 40 µl according to the instructions of the manufacturer (Invitrogen, Life Technologies, Darmstadt, Germany), followed by threefold dilution in 10 mM Tris/HCl, pH 7.5. The qPCR assays were then carried out in a total volume of 25 µl consisting of 1×MesaGreen MasterMix, 5 mM MgCl_2_, Amperase, 300 nM of each primer and 2.5 µl of the cDNA solution, which was derived from 20 ng RNA. Thermal cycling conditions were as follows: 1 cycle at 50°C for 10 min, 1 cycle at 95°C for 10 min followed by 45 cycles of 95°C for 15 s and 60°C for 1 min for amplification, and 1 cycle at 95°C for 15 s, 1 cycle at 60°C for 1 min. The product was than heated from 65°C to 95°C with an increment of 0.5°C/15 s and the plate read for melt curve analysis to check the identity of the amplicon. Each sample was analysed in duplicate. Cycling, fluorescent data collection and analysis were carried out in an iCycler from BioRad Laboratories (Munich, Germany) according to the manufacturer's instructions.

### Western Blot Analysis

Proteins of cell lysates derived from infection assays were separated on 9.5% polyacrylamide gels, transferred to nitrocellulose (Schleicher and Schuell, Dassel, Germany) using a semi-dry blotting procedure and immunostained as formerly published [3].

### Confocal Laser Microscopy

Cells adhering to glass cover slips from the infection assays were triply washed with PBS (taking care to prevent detachment of the cells) and blocked with 2% (w/v) BSA/PBS for 20 min. To differentiate between intra- and extracellular mycoplasma cells the cells were firstly stained with primary antibodies for 45–60 min at 37°C that targeted extracellular antigens of *M. hominis*, washed with PBS, followed by Alexa Fluor-633 conjugated to rabbit anti-mouse IgG (H+L) secondary antibody staining (1∶1000 dilution in 2% (w/v) BSA/PBS; Invitrogen) for 45 min in the dark. Cells were then fixed with 4% (w/v) paraformaldehyde/PBS for 20 min in the dark at room temperature, permeabilized with 0.2% (v/v) Triton-X-100/PBS for 15 min at 37°C, followed by a second antibody, which targeted intra- or extracellular antigens of *M. hominis*, with similar incubation steps to those used for the first staining, but detected by a Cy3-conjugated secondary antibody (1∶1000 dilution in 2% (w/v) BSA/PBS; Dianova, Hamburg, Germany) for 45 min in the dark. For co-localisation studies, cells were fixed and permeabilized as described above and subsequently stained with rabbit mAb MEM-259 (anti-LAMP3; 1∶200) detected by an anti-rabbit Cy2-conjugated secondary antibody (1∶200) followed by a second staining with murine mAb BG11 (anti-OppA of *M. hominis*) detected by an anti-mouse Cy3-conjugated secondary antibody (1∶1000; Dianova). Staining of the cytoskeleton was effected by 30 min incubation with phalloidin conjugated to Alexa Fluor 488 (1∶40 dilution; Invitrogen), staining of membranes with Alexa Fluor 594 Wheat Germ Agglutinin (1∶1000 dilution; Life Technologies) and staining of cell nuclei with 4′,6-diamidino-2-phenylindole (DAPI). Each incubation step was followed by triple washing with PBS. Coverslips were once rinsed with water, positioned with the cell side down on glass slides in Fluoromount-G (Southern Biotech, Birmingham, Alabama, USA) and stored overnight at 4°C in the dark for fixation.

Images were collected using an LSM 510 Meta or an LSM 780 confocal microscope (Zeiss, Jena, Germany). To avoid cross-talk in the detection of the different fluorophores, the multitracking scanning mode was used. Image analysis and processing was performed with ZEN (Zeiss).

### Scanning Electron Microscopy

Scanning electron microscopy (SEM) was performed on sputtered gold coated *M. hominis*-infected HeLa cell samples to evaluate the effects of *M. hominis* infection on the cell shape of HeLa cells using a Hitachi S 3000 N SEM and associated software (Hitachi High Technologies America, Pleasanton, CA, USA).

## Supporting Information

Figure S1 **Recovery of HeLa and** ***M. hominis*** **from each time point of infection.** Total genomic DNA was prepared from cell lysates of each time point of infection, (0 h, 4 h, 24 h, 48 h and 2 weeks post infection) as written in the Material and Method section and simultaneously subjected to TaqMan-qPCR for the detection of human HeLa *gap*-gene (2 copies per genome) and *M. hominis* specific *hit*A-gene (single copy gene). A, bargraph of genome equivalents of HeLa (light grey bars) and *M. hominis* (dark grey). B, bargraph of cell ratios of *M. hominis* to HeLa cells determined by calculation of 2^?(Ct(HeLa)-Ct(M. hominis))^ values from each time point of infection. The data, which are exemplarily shown for the infection assay used for microarray analyses, correspond to those of the other infection assays in reflecting a significant decrement of HeLa cell-colonizing (surface-bound or invaded) mycoplasma cells up to 48 h of infection.
(TIF)

Table S1 **Microarray data set.** Up- and downregulated HeLa cell genes [>1 fold] at 4 h, 48 h or 2 weeks after *M. hominis* infection.
(XLSX)
