# Supplementary figures and images for: Host Cell Responses to Persistent Mycoplasmas - Different Stages in Infection of HeLa Cells with Mycoplasma hominis
Source: PLoS One. 2013 Jan 11;8(1):e54219. doi: 10.1371/journal.pone.0054219 (PMC3543322; doi:10.1371/journal.pone.0054219)

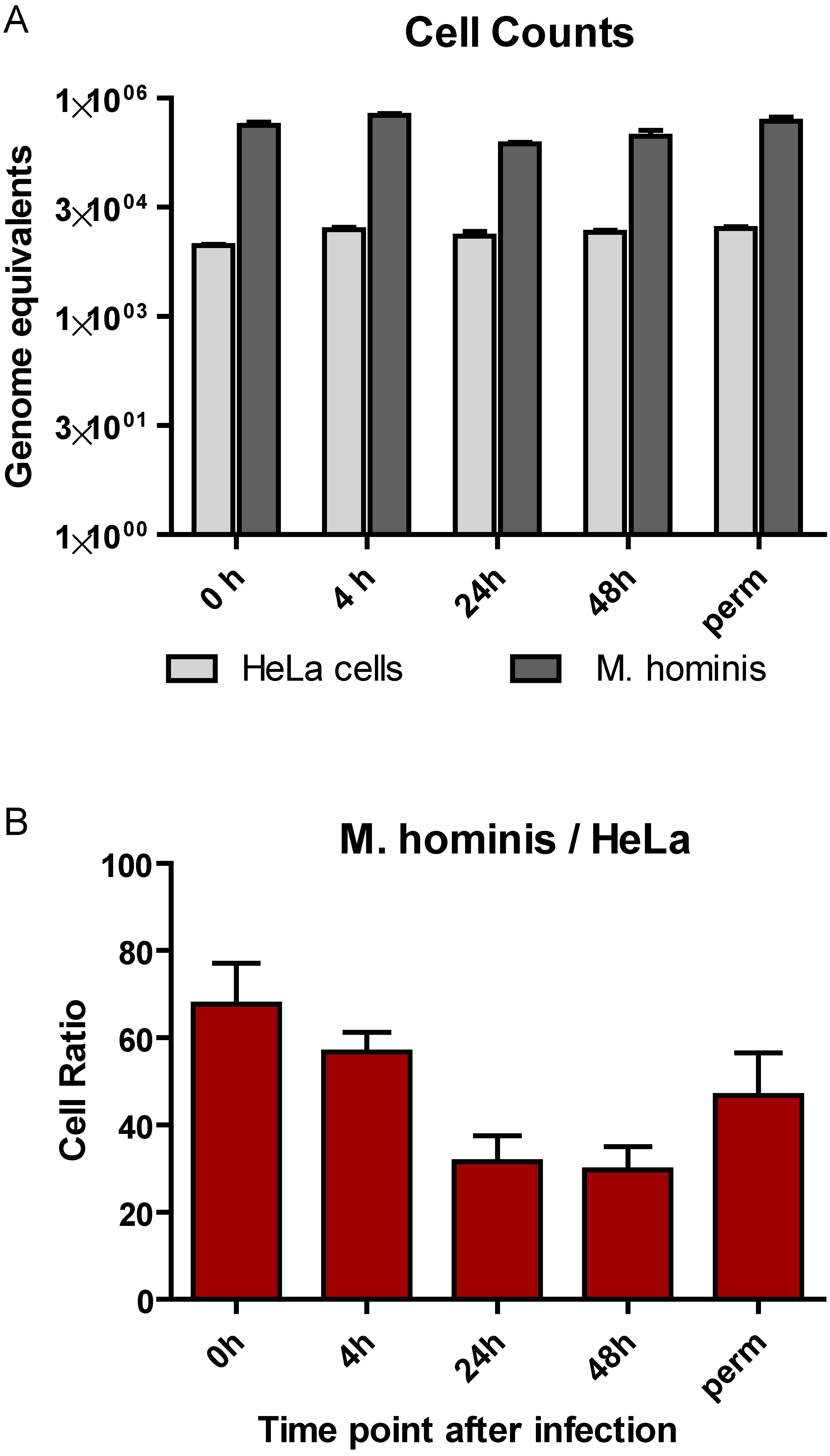

Supplement: Figure S1 — Recovery of HeLa and M. hominis from each time point of infection. Total genomic DNA was prepared from cell lysates of each time point of infection, (0 h, 4 h, 24 h, 48 h and 2 weeks post infection) as written in the Material and Method section and simultaneously subjected to TaqMan-qPCR for the detection of human HeLa gap-gene (2 copies per genome) and M. hominis specific hitA-gene (single copy gene). A, bargraph of genome equivalents of HeLa (light grey bars) and M. hominis (dark grey). B, bargraph of cell ratios of M. hominis to HeLa cells determined by calculation of 2?(Ct(HeLa)-Ct(M. hominis)) values from each time point of infection. The data, which are exemplarily shown for the infection assay used for microarray analyses, correspond to those of the other infection assays in reflecting a significant decrement of HeLa cell-colonizing (surface-bound or invaded) mycoplasma cells up to 48 h of infection. (TIF) [file pone.0054219.s001.tif]
